# Supplementary material for: A sentiment analysis approach for travel-related Chinese online review content
Source: PeerJ Comput Sci. 2023 Aug 23;9:e1538. doi: 10.7717/peerj-cs.1538 (PMC10495948; doi:10.7717/peerj-cs.1538)
Supplement: Supplemental Information 1 [file peerj-cs-09-1538-s001.docx]

Table S1. Experimental results of different dropout

| Dropout | Accuracy | | Precision | F1-score |
| --- | --- | --- | --- | --- |
| 0.1 | | 94.97 | 96.29 | 96.76 |
| 0.2 | | 95.23 | 96.53 | 97.05 |
| 0.3 | | 94.76 | 96.05 | 96.76 |
| 0.4 | | 94.71 | 96.11 | 96.72 |
| 0.5 | | 94.69 | 96.20 | 96.71  96.34 |
| 0.6 | | 94.01 | 94.75 |  |
