# Supplementary material for: A sentiment analysis approach for travel-related Chinese online review content
Source: PeerJ Comput Sci. 2023 Aug 23;9:e1538. doi: 10.7717/peerj-cs.1538 (PMC10495948; doi:10.7717/peerj-cs.1538)
Supplement: Supplemental Information 2 [file peerj-cs-09-1538-s002.docx]

Table S2. Experimental results of different learning rate

| learning rate | Accuracy | | Precision | F1-score |
| --- | --- | --- | --- | --- |
| 0.0005 | | 84.59 | 90.40 | 94.42 |
| 0.00003 | | 94.77 | 96.29 | 96.79 |
| 0.00005 | | 95.23 | 96.53 | 97.05 |
| 0.00007 | | 94.43 | 96.23 | 96.51 |
| 0.000005 | | 94.41 | 96.23 | 96.53 |
|  | | | |  |
