# Supplementary material for: A sentiment analysis approach for travel-related Chinese online review content
Source: PeerJ Comput Sci. 2023 Aug 23;9:e1538. doi: 10.7717/peerj-cs.1538 (PMC10495948; doi:10.7717/peerj-cs.1538)
Supplement: Supplemental Information 3 [file peerj-cs-09-1538-s003.docx]

Table S3. Experimental results of different Convolution filter widths Size

| Convolution filter widths Size | Accuracy | Precision | F1-score |
| --- | --- | --- | --- |
| (2,3,4) | 94.79 | 94.18 | 96.67 |
| (3,4,5) | 95.23 | 96.53 | 97.05 |
| (4,5,6) | 94.97 | 96.03 | 96.89 |
| (5,6,7) | 94.54 | 96.56 | 96.60 |
|  | | | |
